# Effect of Locked-Nucleic Acid on a Biologically Active G-Quadruplex. A Structure-Activity Relationship of the Thrombin Aptamer

**DOI:** 10.3390/ijms9030422

**Published:** 2008-03-24

**Authors:** Laura Bonifacio, Frank C. Church, Michael B. Jarstfer

**Affiliations:** 1 Division of Medicinal Chemistry and Natural Products, School of Pharmacy, University of North Carolina at Chapel Hill Chapel Hill, North Carolina 27599, USA; 2 Departments of Pathology and Laboratory Medicine, Pharmacology and Medicine, Carolina Cardiovascular Biology Center, School of Medicine, University of North Carolina at Chapel Hill Chapel Hill, North Carolina 27599, USA

**Keywords:** aptamer, G-quadruplex, thrombin, locked nucleic acid (LNA)

## Abstract

Here we tested the ability to augment the biological activity of the thrombin aptamer, d(GGTTGGTGTGGTTGG), by using locked nucleic acid (LNA) to influence its G-quadruplex structure. Compared to un-substituted control aptamer, LNA-containing aptamers displayed varying degrees of thrombin inhibition. Aptamers with LNA substituted in either positions G5, T7, or G8 showed decreased thrombin inhibition, whereas LNA at position G2 displayed activity comparable to un-substituted control aptamer. Interestingly, the thermal stability of the substituted aptamers does not correlate to activity – the more stable aptamers with LNA in position G5, T7, or G8 showed the least thrombin inhibition, while a less stable aptamer with LNA at G2 was as active as the un-substituted aptamer. These results suggest that LNA substitution at sites G5, T7, and G8 directly perturbs aptamer-thrombin affinity. This further implies that for the thrombin aptamer, activity is not dictated solely by the stability of the G-quadruplex structure, but by specific interactions between the central TGT loop and thrombin and that LNA can be tolerated in a biologically active nucleic acid structure albeit in a position dependent fashion.

## Introduction

**Figure 1.**
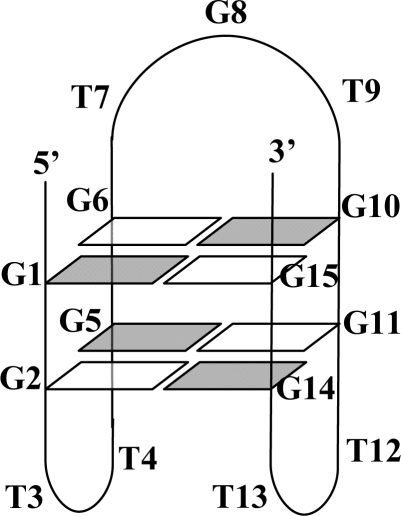
Structure of thrombin aptamer (adapted from Kelly et al [8]). Residues G2, T4, G5, T7, and G8 are individually substituted with LNA analogs in the following experiments.

Nucleic acids that fold into G-quadruplexes have attracted significant attention because of their potential therapeutic applications. G-quadruplexes with anti-HIV [1], anti-proliferative [2], and anti-thrombin [3,4] activities have recently been identified. In 1993, Griffin *et al*. [3,4] used SELEX to identify a 15 nucleotide DNA G-quadruplex with the sequence d(GGTTGGTGTGGTTGG) that demonstrates anti-thrombin activity. The thrombin aptamer offers several potential clinical advantages over more traditional drugs for use in anticoagulation. For example, the short half-life of the thrombin aptamer makes it rapidly reversible. Further, this targeted oligonucleotide displays high specificity, which decreases the potential for untoward effects. The interaction between thrombin and the DNA thrombin aptamer has been characterized by x-ray crystallography and NMR, and binding was shown to occur at exosite 1 (the anion binding exosite) [4,5]. This binding mechanism confers yet another advantage to the thrombin aptamer; because it binds at thrombin exosite-1 (rather than the active site) the thrombin aptamer is able to inhibit both free and clot-bound thrombin [6]. Structurally, the thrombin aptamer folds into an intramolecular, chair-like G-quadruplex with two stacked G-tetrads and three flanking loops (Figure 1) [7]. Despite this seemingly well-defined structure, several unique folding topologies of the thrombin aptamer are possible [8]. To what extent these contribute to the biological activity of the thrombin aptamer is not known and can only be accurately addressed if specific folding topologies can be produced.

Recently, we found that incorporating a single locked nucleic acid (LNA) residue into a DNA G-quadruplex can influence its structure [9]. LNA are 2′-*O*-4′-*C*-methylene-linked ribonucleotide nucleic acid analogues that bind with increased affinity to DNA and RNA oligomers due to a forced 3’endo conformation of the glycosidic residue [10,11]. LNA-containing oligonucleotides have been utilized for various applications including defining the HIV-1 PPT recognition by HIV-1 RT [12], as probes in developing real-time PCR protocol for genotype analysis [13], and to increase efficiency in targeting antisense oligonucleotides with various ribozymes [14]. In general, LNA has been most extensively explored to increase the affinity of oligonucleotides for DNA and RNA targets [15]. The bicyclic structure of LNA forces the sugar to be in the C3′-*endo* conformation, and nucleotides with a C3’-*endo* conformation prefer the glycosidic bond to be in the *anti* configuration. Because G-quadruplexes have very specific requirements for the sugar pucker and glycosidic bond configuration [16], we reasoned that a strategically placed LNA would influence the G-quadruplex folding topology of the thrombin aptamer potentially affecting its activity. As we were completing our work, the effect of extensive LNA substitution on the structure of the thrombin aptamer was reported [17], but the biological effect of only a single LNA at the terminus of the thrombin aptamer was reported [18]. Here, we examined the effect of LNA substitutions at several positions in the thrombin aptamer on its activity.

## Results and Discussion

### Biophysical Characterization of Thrombin Aptamers

**Figure 2.**
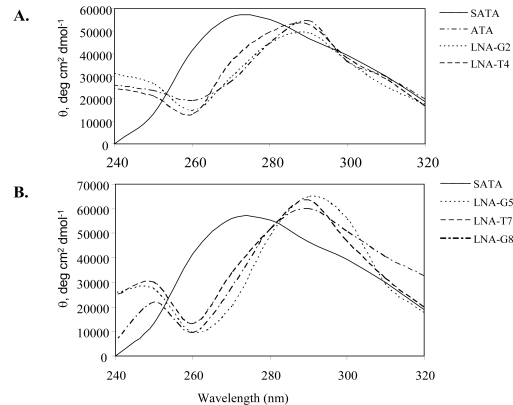
CD spectra of thrombin aptamer and LNA-containing thrombin aptamers. CD spectra were obtained using 5 μM oligonuceotide in 10 mM Tris buffer, pH 7.5, containing 1 mM EDTA and 50 mM KC1. ATA is a scrambled aptamer (see Experimental Procedures).

In a previous communication, we reported the finding that LNAs can affect the folding topology of G-quadruplex DNA [9]. This prompted us to examine the ability of LNAs to influence the activity of a biologically active G-quadruplex. We examined the G-quadruplex forming thrombin aptamer, d(GGTTGGTGTGGTTGG), containing single LNA substitutions in several positions, which are indicated in Table 1. We first determined the effect of the LNAs on the folding topology of the thrombin aptamer. Using CD spectroscopy, we found that the thrombin aptamer and each LNA-containing derivative produced a strong, positive CD absorbance at 295 nm with a minimum at 260 nm, indicative of an anti-parallel quadruplex (Figure 2).

Upon heating to 95 °C, this signal disappeared suggesting a thermally labile structure as expected (data not shown). By contrast, the scrambled aptamer, SATA, displayed a spectrum consistent with single-stranded, random coil DNA with a strong positive absorbance from base stacking at 275 nm and a minimum at 238 nm.

**Figure 3.**
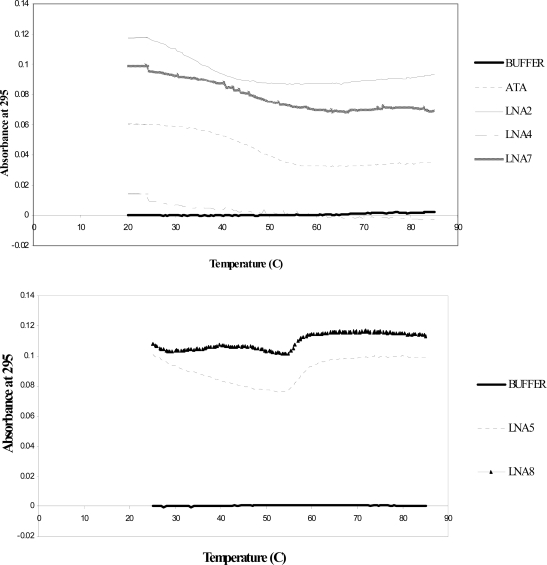
UV melting curves for LNA-substituted and unsubstituted thrombin aptamers.

The thermal stability of the thrombin aptamer and the LNA-substituted aptamers was determined using UV melting curves in buffer containing K^+^. The LNA substitution had either a moderate stabilizing or destabilizing effect on the folded structure, depending on the position of the LNA in the thrombin aptamer sequence, though we were unable to measure the melting temperature for the aptamer with LNA in position T4, presumably because it is particularly unstable (Table 1). Interestingly, the melting curves of the LNA-G5 and LNA-G8 thrombin aptamers were hyperchromic instead of the expected hypochromic change (Figure 3).

Combined with the CD spectroscopy data, the biophysical characterization of the thrombin aptamer and LNA-containing derivatives confirmed the presence of an anti-parallel G-quadruplex in the LNA-substituted aptamers. We also observed both stabilizing and destabilizing effects of the LNA, which is consistent with our previous report that LNA substitution demonstrates a position dependent effect on the stability of G-quadruplexes [9]. Interestingly, we did not observe a change in folding topology of the thrombin aptamer to a parallel quadruplex, which would generate a CD spectrum with a maximum at 260 nm. This contrasts our findings with a telomeric G-quadruplex, for which LNA could induce folding into a parallel structure [9]. Perhaps the thrombin aptamer is incapable of producing a parallel G-quadruplex. One recent report shows that LNA at position 15 of the thrombin aptamer results in formation of an anti-parallel G-quadruplex, consistent with our interpretation [18]. Further analysis of the substituted aptamers by NMR will be required to determine their fine structure.

### Biological Activity of Thrombin Aptamers

**Figure 4.**
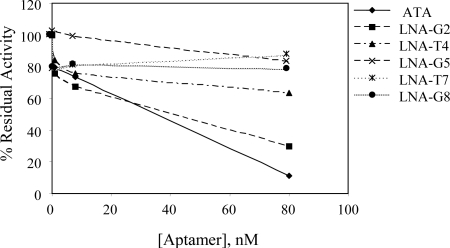
Thrombin inhibition by thrombin aptamers as assessed by a fibrinogen clotting assay. Inhibition of thrombin aptamers was determined by measuring the residual thrombin activity after incubation with the indicated thrombin aptamer. Data are representative of at least five data sets and the standard deviation between data sets was <5%.

The effect of LNA substitution on the biological activity of the thrombin aptamer was determined using two different assays: fibrinogen clotting assays and heparin template curve assays, in which heparin cofactor II (HCII) and anti-thrombin III (ATIII) thrombin-inhibition assays are performed in the presence of heparin. Previously, the quadruplex structure of the aptamer was shown to be essential for activity, as the scrambled aptamer used during characterization steps was shown to be inactive [19]. Generally, the un-substituted thrombin aptamer exhibited a greater capacity for inhibiting fibrinogen clotting activity than did the LNA substituted derivatives (Figure 4). LNA substitutions at G5, T7, or G8 dramatically reduced fibrinogen clotting activities, whereas LNA substitutions at G2 and T4 only had slightly decreased inhibitory activity when compared to the un-substituted thrombin aptamer. These results were not expected because, in general, the stability of a biologically active G-quadruplex coincides with activity. Interestingly, the LNA-G2 thrombin aptamer was one of the least stable quadruplexes (as determined by thermal melting curves) but demonstrated activity equal to the control aptamer. By contrast, LNA-G5 and G8 thrombin aptamers were more stable than the control thrombin aptamer but were less active with respect to inhibition of fibrinogen clotting activity.

**Figure 5.**
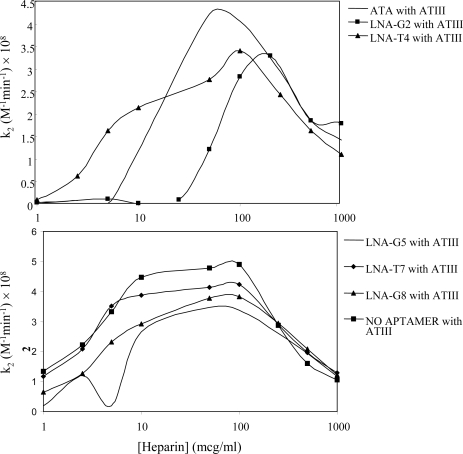
ATIII-heparin inhibition of thrombin in the presence of thrombin aptamers. The rate of thrombin inhibition by ATIII was determined using final concentrations of 1 nM thrombin, 10 nM ATIII, 5 nM aptamer, and increasing concentrations of heparin from 0.1 to 10,000 μg/ml (6.7 – 66,667 nM).

It is possible that LNA substitutions could influence the structure of the thrombin aptamer and change the nature of aptamer-thrombin interaction. To test if the LNA-substituted aptamers bound to a different site on thrombin, we utilized heparin template curve assays in the presence of serpins that are thrombin inhibitors. Two serine protease inhibitors (serpins) were used for these assays, HCII and ATIII. Both serpins were examined because HCII requires thrombin exosite-1 as part of its inhibitory mechanism, while ATIII requires thrombin exosite 2 but not exosite-1 for its inhibition of thrombin in the presence of heparin. Thus, binding of the thrombin aptamer should interfere with binding of HCII to thrombin but not perturb the binding of ATIII. We would expect a similar effect on the rate of thrombin inhibition by each serpin if the LNA-substituted aptamers bind exosite-1 in the same manner as the un-substituted thrombin aptamer. Consistent with this expectation, we found that the presence of the substituted and control aptamers did not influence ATIII-mediated thrombin inhibition in the presence of heparin (Figure 5).

**Figure 6.**
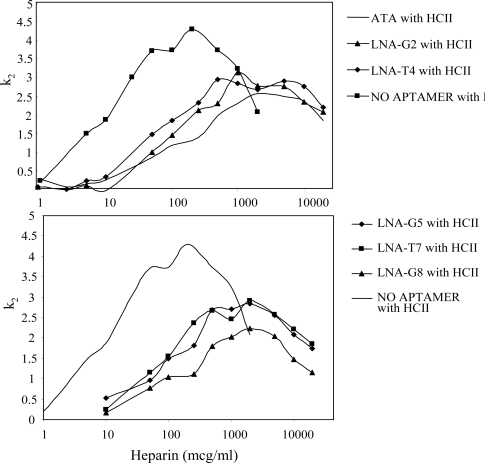
HCII-heparin inhibition of thrombin in the presence of thrombin aptamers. The rate of thrombin inhibition by HCII was determined using final concentrations of 1 nM thrombin, 10 nM HCII, 5 nM aptamer, and increasing concentrations of heparin from 0.1 to 10,000 μg/ml (6.7 – 66,667 nM).

In contrast, we found that a greater concentration of heparin was required to achieve the maximal *k*_2_ for the thrombin inhibition reaction when HCII was in the presence of either the substituted or control aptamers (Figure 6). This suggests that the LNA substituted and control thrombin aptamers both bind to exosite-1 and do not interfere with serpin binding to exosite 2. We were surprised to find that the aptamers with LNA substitutions at positions G5, T7, or G8 did not inhibit fibrinogen clot formation of thrombin, but did affect HCII-mediated thrombin inhibition in the presence of heparin. Collectively, the results suggest that these LNA-containing aptamers do bind to thrombin and interact specifically with thrombin exosite-1, but they also suggest subtle changes either in binding affinities or their ability to bind unique thrombin exosite residues. The lower activity of the LNA-aptamers suggest that subtle changes in the aptamer binding to thrombin exosite-1 contribute to anti-thrombin activities as demonstrated here with two different thrombin exosite-1-dependent reactions, fibrinogen clotting and HCII/heparin-catalyzed thrombin inhibition.

The disparity between LNA-induced effects on biological activity and stability of the aptamers is likely due to subtle local structural effects rather than global structural effects or effects on thermal stability. It appears that the location of the LNA substituted bases within the quadruplex structure dictates the biological activity but not necessarily thermal stability. One possibility is that the conditions used in the biological assays stabilize the modified aptamers. However, even if this were the case, it does not seem likely that the stabilizing effect would be sequence specific in a manner not reflected by the thermal stability studies. It is more likely that substituting LNA into position G5, T7, or G8 interferes directly with binding between thrombin and the aptamer. Of note, T4 is positioned in one of the two minor T-loops of the aptamer’s G-quadruplex structure (Figure 1). Hydrogen bonding and base stacking interactions between T4 and T13 in the aptamer have been shown to be integral for the aptamer’s ability to inhibit thrombin. For example, a T to A substitution at T4 resulted in a loss of thrombin inhibition [23]. It is therefore possible that a substitution of LNA into position 4 could serve to attenuate thrombin inhibitory activity by exerting a destabilizing effect on the aptamer. As mentioned earlier, T7 and G8 LNA-substituted aptamers both showed relatively weak inhibition of thrombin and are located in the major T-loop of the quadruplex. Perhaps the decrease in thrombin inhibition activity of LNA5, LNA7, and LNA8 is due to interference of specific protein-DNA interactions.

In summary, we have demonstrated that LNA can substitute for normal DNA in a biologically active G-quadruplex. These data can be combined with those published by Virno *et al*. [17], to generate a structure activity relationship for the thrombin aptamer. Virno *et al*. showed that substitution with LNA of all residues, all guanosine residues, or only the G1 residue resulted in inactive aptamers, whereas substitution at G15 resulted in partial activity even though the G15 substituted structure folded into a stable quadruplex. Combined with our results, we conclude that for the thrombin aptamer, overall G-quadruplex stability is not the major contributor to biological activity, which is unexpected. Instead, the local structure of the TGT loop appears to dominate activity, presumably due to its direct interaction with thrombin at exosite-1.

## Experimental Procedures

### Materials

#### Oligonucleotides

The thrombin aptamer d(GGTTGGTGTGGTTGG) was obtained from Integrated DNA Technologies (Coralville, IA). Thrombin aptamer analogs containing individual LNA substitutions in positions G2, T4, G5, T7, or G8 were provided by Proligo (Boulder, CO). All aptamers were purified by denaturing polyacrylamide gel electrophoresis. Gels were prepared with 20% acrylamide, 7 M urea, and 1×TBE (89 mM Tris, 89 mM boric acid, 2 mM EDTA, pH 8.5) and 1× TBE was used as the running buffer. Oligonucleotide bands were visualized by UV shadowing and extracted from the gel by the crush and soak method. Briefly, gel slices were crushed with a glass rod and extracted twice with TEN (10 mm Tris-HCl, pH 8.0, 1 mm EDTA, and 200 mM NaCl). Purified aptamers were concentrated by ethanol precipitation and resuspended in TE (10 mm Tris-HCl, pH 8.0, and1 mm EDTA). Aptamer concentrations were determined by UV spectroscopy using an extinction coefficient of 143,300 M^−1^cm^−1^ and were determined to be single sequences by PAGE analysis.

#### General Materials

HNPK (20 mM HEPES, 145 mM NaCl, 5 mM KCl, 0.1% polyethylene glycol 8000, 0.02% NaN_3_ at pH 7.4) was used as the buffer in all coagulation studies. TEK (10 mM Tris pH 7.5, 1 mM EDTA and 50 mM KCl) was used as the buffer for thermal stability analyses and CD experiments. Thrombin solutions were prepared from a stock solution of human α-thrombin that was purified as described previously [19].

### Methods

#### Fibrin Clotting Assays

Continuous kinetic assays were performed in standard U-bottom, ovalbumin (OVA)-coated, 96 well microtiter plates at room temperature. Solutions of thrombin, fibrinogen, and aptamer were prepared in HNPK. In individual wells, thrombin (2 nM) was incubated with increasing amounts of aptamer (1 nM, 10 nM, 100 nM) for 1 min. After 1 min, fibrinogen (4 mg/ml) was added and clotting was measured by UV absorbance and reported as residual thrombin activity [20]. Each reaction was performed in duplicate and negative controls included duplicate samples containing no aptamer, and reactions containing a scrambled aptamer d(GGTGGTGGTTGTGGT).

#### Heparin Template Curves

HNPK was used as the buffer for these assays which were performed with 1 nM thrombin, 10 nM serpin [heparin cofactor II (HCII) or anti-thrombin III (ATIII)], 5 nM thrombin aptamer, 150 μM tosyl-Gly-Pro-Arg-p-nitroanilide (GPA), and increasing concentrations of heparin (0.6–20,000 μg/ml). Thrombin was added 15 seconds before the addition of the substrate GPA, and the reaction was quenched 10 min later by the addition of 50 μL of 50% acetic acid. Reactions with each concentration of heparin were performed in quadruplet and corresponding controls containing no HCII or ATIII were performed in duplicate. After reactions were quenched, plates were covered with microplate adhesive film and spun at 2800 rpm for 15 min at rt. 100 μL of each supernatant was removed and analyzed by UV at 405 nm using a Thermomax microtiter plate reader (Molecular Devices). Rate constants were calculated as described by Ciaccia *et al* [21] and are reported as second-order rate constants of inhibition (M^−1^ min^−1^) and were plotted vs heparin concentration.

#### Thermal Stability Analysis

Thermal stability of the thrombin aptamer and LNA-substituted aptamers was determined by UV melting curves obtained on a Perkin Elmer UV/Vis Spectrophotometer Lambda 20 using a PTP-6 Peltier System to control the temperature. Aptamers were prepared as 5 μM samples in 1X TEK in a final volume of 400 μL. Samples were heated from 20–85°C, at 1°C min^−1^, and the absorbance at 295 nm was recorded [22]. Absorbance was measured for both melting and reannealing of the aptamers. Melting temperatures were calculated from the first derivative of the duplicate absorbance versus temperature curves.

#### Circular Dichroism

Oligonucleotides (5 μM) were annealed by heating samples for 5 min at 95°C then slow cooling (1°C min^−1^) to room temperature in 400 μl 1X TEK buffer. CD spectra were recorded on an Applied Photophysics Pistar-180 Circular Dichroism spectrophotometer equipped with a Peltier temperature controller. For each sample, spectral scans were collected over the wavelength range 200–350 nm at 25°C in a 1 mm path length cell at a scanning rate of 20 nm min^−1^. Separate spectra were obtained at 95°C. Three scans were averaged for each sample, and the scan of the buffer alone was subtracted for each sample. CD spectra were collected in units of millidegrees versus wavelength and normalized to the strand concentration and number of residues.

## Figures and Tables

**Table 1.** Thermal stabilites of G-quadruplexes used in this study. (a) Thermal stability was determined by using the first derivative of the absorbance versus temperature curve (b) The first oligonuceotide is the control, un-substituted thrombin aptamer (ATA). Subsequent oligonuceotides contain LNA at the locations indicated by the listed base. (b) The melting temperature could not be determined for this aptamer.

Name	dA/dT (°C)^a^	Oligonucleotide^b^
ATA	48.1	5′d(GGTTGGTGTGGTTGG)
LNA-G2	33.48	5′d(-G----------------)
LNA-T4	n.d.^b^	5′d(---T---------------)
LNA-G5	50.7	5′d(----G--------------)
LNA-T7	43.4	5′d(-------T------------)
LNA-G8	50	5′d(----------G---------)
